# The good, the bad and the ugly – a Swedish qualitative interview study about the landscape of meaning-imbued, exercise-related physical pain, as experienced by ‘normal’ gym-users

**DOI:** 10.1186/s12889-024-18623-6

**Published:** 2024-04-25

**Authors:** Pelle Pelters

**Affiliations:** Department of Education, University of Stockholm, Stockholm, SE-106 91 Sweden

**Keywords:** Pain, Exercise, Gym-users, Gym, Health promotion, Qualitative content analysis

## Abstract

**Background:**

The gym is a well-known place for health promoting or rehabilitating exercise whose availability to all is regarded significant for people’s personal health work and the public’s health. In this context, physical pain is usually discussed as something negative that people seek to dispose of. However, certain painful experiences appear to be an appreciated part of the gym experience. To investigate this seemingly contradictory landscape of meaning-imbued physical pain, the study aims to explore the different kinds of physical pain present at the gym and their significance for exercising, as experienced by ‘normal’ gym-users.

**Methods:**

24 semi-structured in-depth interviews with active, dedicated, reasonably healthy (= normal) adult gym-users have been analyzed using qualitative content analysis from a hermeneutical stance.

**Results:**

Participants differentiate between three kinds of physical pain: the good pain of enhancement (often connected to muscle soreness and effort burn), the bad pain of impediment (primarily related to acute damage) and the composite, neutral pain of acceptance (potentially linked to all pains).

**Conclusion:**

When pursuing the goal of personal health development, normal gym-users argue that exercising at the gym means to expose yourself to pain and to do so willingly, even longingly. Refusing to share this understanding may diminish people’s chances to occupy the gym space and, hence, reduce their chances to promote their health.

## Background

Overhearing the cheery exclamation *“Sore muscles, that’s what we like!”* by one of my own fitness instructors and recognizing my own reaction (“*Wait, why on earth would I want that*?”) sparked off the following study about the role of different kinds of pain in the gym.

In the background section, I will first address existing research about the gym culture in general before providing an overview on physical activity and pain within the realm of the gym.

### Gym culture

The present study is situated in the gym, which provides a space for exercising ‘on demand’ in organized classes or at a self-selected time. This venue may be regarded as a popular, even iconic environment and location for exercise that is health-motivated [1–3]. In this regard, the gym may be understood as a signifier of healthy lifestyles, in sync with dominant health discourses and health norms, which regular gym-users appear to predominantly follow [4, 5]. Their practice usually aims at promoting one’s health but balances potentially on the verge of becoming “an unhealthy obsession” [6, p. 219, cf. also 7].

At least of the same importance is the orientation of gym exercise towards gaining fitness. “Fitness has become the overall concept used when referring to health clubs and fitness franchises, and has thereby turned into a popular movement (…) [that is] highly individualised and personal” [5, p. 8] and strongly associated “with values such as health, youth and beauty” [5, p. 9].

Finally, gym exercise is directed towards shaping the body [5, 8, 9] that then may function as a lifestyle marker and represent the good life [10]. The aimed-for, idealized body ‘in shape’ is no longer the overly muscular but the well-defined body that looks young, energetic, fit, attractive and impressive [5, 9], a body that by representing fitness is assumed to represent health, too [11].

The gym of the present-day “fitness revolution” [5, p. 8] is supposed to be a place for everyone [5] with a gym culture that is idealized as inclusive, albeit being more inclusive to some than others. It clearly privileges the masculine, white, middle-class person with “sufficient capital to be able to consume it, in terms of available leisure time, economic capital, geographical proximity” [11, p. 4]. In this gym, the ‘old’ core values of bodybuilding are still at play, i.e. intense exertion-oriented muscle training, asceticism and competition [5, 9]. These values, in line with a fitness-oriented health discourse and body ideal, contribute considerably to the prevalence of a so-called “*no pain, no gain* culture” [9, p. 119], which celebrates youthfulness [9]. This culture has been described as globalized, heavily commercialized and characterized by a McDonaldisation, i.e. it represents a highly standardized, regulated and predictable enterprise that promotes a homogenized global body ideal linked to self-regulation and self-government [5, 12]. However, gym cultures may also vary depending on local contexts and expectations [4].

When it comes to Sweden, this local cultural variation has of yet not been clearly addressed in research although some clues regarding its specific features can be derived. On the backdrop of the high esteem for gender equality in Sweden, there are indications that the common masculine norm is potentially challenged and transgressed, enhancing the status of both female fitness professionals [4] as well as the scope of acting and being for regular female gym-users. Here, the fact that in northern European countries women’s fitness participation outnumber that of men [13] may serve as a supporting argument for this assumption. Whereas for women, a more diverse body ideal may include moving form a focus on esthetics to strength performance [14], all genders may be exposed to a more relaxed approach to exercising and dieting by fitness professionals [8]. This picture, however, is far from clear-cut [8] and distinct gendered spatial orientations can still be found [11].

In addition, a potentially relevant recent aggressive exercise and fitness competition trend has been observed in Sweden [7] and statistics show that “fitness is more popular in Nordic countries” [13] compared to the rest of Europe. This may reflect an advanced focus on the independent, self-realizing and self-challenging individual in Sweden (as also indicated by the World Value Survey, 15, describing Sweden as characterized by high levels of secular-rational and self-expression values). On the whole, Sweden may be described as a full-blown neoliberal Western society, with an individualized health care system and an increasing emphasis on a health promotion that focuses on gym and fitness exercises [16]. In this space, specific practices, attitudes and feelings related to health, fitness and the body are constructed and learned in a necessary ‘gym work’, in which pain represents an integral part [17, 18]. The gym in general and the Swedish one in particular may hence be regarded a place in which physical pain thrives and concerns all gym-users.

### Physical activity, pain and the gym

Physical activity (PA), especially exercise is considered an unequivocal cornerstone for promoting health due to its “significant health benefits for hearts, bodies and minds” [19]. For at least a decade, a considerable part of organized recreational sports in Sweden has been directed to promoting health in a wider sense, stating goals of sports such as providing a “possibility for physical, mental, social or cultural development” [20, p. 7/8]. However, a considerable part of populations in western societies (approximately 20% of the Swedish population for example, 1) rarely or never exercise, imparting significance to questions about beneficial conditions for physical work-out activities.

These questions are usually addressed with regard to personal motivations to exercise [see e.g. 21, 22]. Primarily physical pain is presumed to affect this motivation to engage in PA. It is usually addressed as a barrier to be physically active or as something that requires coping, implying this is not an easy task [e.g. 23–25]. Common (bio-)medical or public health research moreover often focus on how target groups with certain risks or ailments can be motivated to become physically active and alleviate pain [e.g. 26, 27]. Pain is hence ascribed a negative connotation despite its positive function as a warning system for overstrain and injury [28, 29]. This negative connotation is also highlighted in descriptions of other academic provenances intending to capture its essential features. An example is pain’s depiction as “a way of finding oneself in the world that typically leads to certain emotions of the negative type: frustration, irritation, anger, fear, sadness, self-pity or even loss of hope and trust in others” [30, p. 543]. This negatively connoted pain has often been advocated as the prime example for how the usually absent body re-appears into a person’s experience and demands attention in a negative fashion as a so-called dys-appearance [31].

But the body may also reveal itself to one’s experience in a positive way in terms of a eu-appearance [31]. There are indications that pain may make such a positive entry into experience in gym exercise. Positively connected pain in the gym has been mentioned and, in some cases, explored in more depth in previous research, of which a considerable part, however, has been conducted in the context of bodybuilding, CrossFit or ‘hardcore’ gyms and often reveal (hyper-)masculinely connoted and competitive environments and practices.

A study about the construction of healthy bodies in the bodybuilding subculture, for example [32], describes the so-called erotics of the gym – “the sensual bodily pleasures of anaerobic exercise” [32, p. 348] – as an embraced and enjoyed culture of pain. Paradigmatic for these pleasure-producing practices is ‘the pump’, which has been famously described by Arnold Schwarzenegger as equaling sex: “blood is rushing into your muscles (…) it’s really tight like somebody is blowing air into your muscles (…) It’s as satisfying to me as coming is, you know, as having sex” (quotation in [32, p. 345]). However, enjoying the pump and other “non-injurious, self-inflicted and self-controlled `pain’” connected to the pump [32, p. 345, cf. also 33] is something that has to be learned, a part of being socialized into the bodybuilding subculture and attaining competence and confidence as inaugurated bodybuilder. The enjoyment consists hence in feeling empowered but may also be experienced as a rush of positive sensations similar to drugs.

Another study investigated the role of CrossFit coaches in the process of becoming a CrossFit athlete [34], in which experiences of excruciatingly intense, vomiting-inducing work-outs are important for neoliberal subjects to realize and constantly improve their ‘fit’ and ‘healthy’ bodies. The enjoyment lies here in the individual’s capacity to stand the pain and embrace it for the sake of personal transformation. This positively-connoted pain is experienced during work-outs, as connected to anaerobic exercise. It is normalized by CrossFit coaches as an obvious part of the experience, despite them voicing concerns about the bad pain of (potential) injury as well. As in the bodybuilding study, the endurance of anaerobic pain is connected to achievement and self-mastery – depicted as a “‘no pain, no gain’ ethos” [34, p. 1445] – although it does not appear to be understood as equally satisfying or even sexualized. This notion of at times painful bodywork as both craft and graft, signifying the production of muscular male bodies within the consumptive sphere of the gym has also been depicted in ethnographic studies in ‘hardcore’ gyms [35].

Only one study was identified that focused on the significance of pain for ‘mainstream’ gym-users. In this case, the emphasis was put on sore muscles, which appeared as a clearly positive, desired pain. It was even addressed as “glorious pain” [17], characterized by enjoyable and rewarding qualities. Sore muscles are clearly experienced as something that should be pursued and give reason to experience pride. Similar to the appreciation of anaerobic pain in body building [32], praising sore muscles proved to be the result of a learning process, which affects and enhances the process by which people attending a gym change from novice to experienced gym-user.

While these studies focus pain directly, a considerable number of studies, investigating regular, normal gym-users, mention pain rather in passing. Although in one case, exercise-related pain in the gym has been experienced as “seductive” [36, p. 186], it is usually depicted as something that rather needs to be overcome or pushed-through, indicating some experience of self-mastery and a necessity regarding the successful realization of creating a certain body [37–39] or the self [36, 40]. In fact, gym-users on every level of ambition are described to expect exercise to be painful, implying that, for them, it is only true exercise if it involves a certain amount of painful discomfort [8] – as is represented in the description of the gym as representing a ‘*no pain, no gain*’ culture [9]. Pain may thus basically be regarded as a sign of “proper exercise” [41, p. 40]. However, another study indicates that certain, albeit not all pains may be pleasurable, leaving the gym-user with the task “to know which they are and how to stimulate them” [41, p. 40] in order to get absorbed in the exercise and focus on one’s goal.

It can be concluded that even if negatively connoted pain in the context of rehabilitation and prevention is assigned an important role in this regard [42, 43], previous research indicates that it is primarily positively-connoted pain, which is connected to understanding exercise as a transformative experience. The reported attitude of regular gym-users towards this positive role of pain is, however, not unambiguous. Whereas Arlie and colleagues [44] for example report that “[k]een exercisers would appreciate the saying ‘no pain, no gain’”, Frew and McGillivray [45] depict fitness professionals’ frustration with clients not showing sufficient commitment “to go through the pain” [45, p. 170] in order to gain results, indicating that this appreciation is not pervasive, as well as holding clients responsible for the development of their performance [cf. also 34].

From this brief overview it can be subsumed that:

- Most of the above-mentioned studies focus only on a fraction of potentially experienced physical pains in the gym and do not give a comprehensive overview of pains, their role and significance for exercising as experienced and understood by gym-users.
- Studies targeting normal gym-users often only allude to the topic and do rarely investigate the role of positively-connoted pain for this group’s exercising practice in-depth.

Hence, a dearth of research in this respect is stated. It is therefore deemed suitable and important to study this pain landscape in a detailed and comprehensive way. The aim of the study was therefore to explore the different kinds of physical pain present at the gym and their significance for exercising, as experienced by ‘normal’ gym-users.

## Theoretical resources

This study applies the perspectives of norm-criticality [46] as informing this study’s aim and design. Moreover, representations of health promotion [6, 47, 48] in conjunction with thoughts on the role of fitness in liquid modernity and cultural understandings of pain [49] operate as analytic instruments in the discussion of this study’s results.

The *norm-critical perspective* redirects attention from (often explicit) individual motivations to that which is normal, often unquestioned, implicit. Directing attention to normality (instead of deviance) allows for directly targeting socially constructed normative expectations that exist in a certain environment (such as the gym) regarding how elements of this environment (such as pain) should be understood, dealt with and felt towards of those intending to feel at ease in the environment (such as ‘normal’ gym users). Hence these normative expectations function as demands, which regular dwellers in this environment are supposed to assimilate to and in doing so confirm – they regulate the practice, attitude and identity of ‘normal’ gym-users [cf. 17, 7]. This perspective has therefore motivated the choice of ‘normal’ gym-users as participants. Coming, moreover, from an understanding of pain as a common, yet often implicit element of the gym culture (cf. the talk about “the *No pain, no gain* culture” in the gym, in [9, p. 119]), norm-criticality’s focus on highlighting this very type of implicit normative expectations has also informed the exploration of different kinds of usually unquestioned, self-evident and often even unconsciously present physical pain at the gym and their significance for exercising, which this study aims at.

In addition, assuming that pain is a self-evident, even desirable part of gym exercise [cf. 17, 41] generates questions as to who is and who is not allured by the gym and a commitment to this type of presumably health promotive and allegedly non-discriminatory practice. Hence, the norm-critical perspective also contributes to questions for interrogating the results of the study with regard to practical implications, which will be addressed in the corresponding sub-section of the discussion.

The analytic discussion of the relation between pain and exercise will, however, be mostly informed by cultural concepts of pain [49] as directly related to the studied topic as well as concepts concerning health promotion and fitness [6, 50, 51], which significantly frame exercising at the gym.

*Health promotion* can be understood as “the set of discourses and practices concerned with individual behaviours, attitudes, dispositions or lifestyle choices said to affect health” [6, p. 219]. It may be considered a ritual, i.e. a means for meaning-making and providing practical rules regarding “the conditions and possibilities for a ‘good life’” [6, p. 220]. As such, health promotion reflects the contradiction and ambiguity between societal demands for immediate pleasure and release inherent to consumption on the one hand and for the disciplined control and delayed self-realization of production on the other [6]. This Crawfordian binary of pleasure/consumption and control/production will be analytically applied to gain a deeper understanding of exercise as one of health promotion’s important signifying practices permeated by implicit expectations about what exercise-related pain ‘is’ [cf. e.g. 52].

Moreover, by aiming at enabling “people to increase control over, and to improve, their health” [48, p. 1] health promotion promises an actualization of people’s “fullest health potential” [48, p. 1]. Hence, the prospective concept of potential indicates here a never-ending process of becoming in the name of health that resembles the one, which Bauman assigns to fitness [51]. Its unattainable endpoint of “complete physical, mental and social well-being” [48, p. 1] equals the state of complete fitness which is just as out of reach, “as one can always become faster, stronger, or more powerful” (35, p. 230, even if Bauman states otherwise regarding the possibility to be satisfied with one’s health and argues against equating fitness with health). As increasing fitness is about increasing the body’s capacity to experience new sensations, consume pleasure and be able to participate and contribute to the individualized consumer society devoid of traditional support networks in *liquid modernity* [50, 53], it meets the good life envisioned by the ritual of health promotion [6].

The unattainability of both may then contribute to anxiety and insecurity in the individual, a constant feeling of latent failure and inadequacy regarding the task of continuous self-formation and a life well lived, for which health and fitness are considered requirements and the body an ambivalent site of consumed pleasures and incognizable dangers [50]. The production of bodies in the gym may in this respect be understood as a prerequisite for consumption, but might – as “production within consumption” [35, p. 220] – also become a part of it, as the globalized fitness industry [4] provides the productive means, which people by way of consumption employ in their individual health/body work [35]. In addition, neoliberal just as consumer societies put specific emphasis on the free choice and responsibility of the prudent, self-governing, self-forming citizen. With regard to health promotion, “personal issues, such as health-related lifestyle behaviours, [are rendered] into moralistic issues” [47, p. 103] to be solved by the individual. It is against this backdrop that the individual is assigned the task to solve the predicament between consumption and production [6] as well as the unending task of continuously becoming more and more fit and healthy [51] – and is held accountable (not least by themselves) if failing. Neoliberal ideals appear to be especially embraced and realized in the gym context [54]. The notion of fitness/health as a never-ending, potentially anxiety-provoking process, in which production and consumption are intertwined, will be applied in the discussion, not least to critically explore the potential of pain to ‘reverse polarity’ (i.e. pains that are experienced as good might become bad pains).

Pain will otherwise be addressed with regard to its double cultural frame of reference in western societies: On the one hand, pain is rejected and viewed as an unproductive, even deconstructive threat, inducing efforts of (medical) pain avoidance. On the other hand, an engagement with pain may be understood and sought as transcending and positively productive of cultural meanings and identities. This understanding can be traced back to the meaning of pain in (monotheistic) religions [49]. Whereas the notion of an unproductive pain precedes the one of a productive, transcending pain in society, both understandings exist and will be applied in the analysis.

## Method

### Data collection – participants and study design

Participants of this qualitative interview study consisted of adult gym-users aged 27–76 who did not have ambitions to compete. The latter criterium (no competitors included) is motivated by the focus on ‘normal’ gym-users, which in this case means that participants should not identify themselves as for example ‘body builder’, ‘weightlifter’ or contestant in CrossFit, strength athletics or similar competitions as these people might have learned to normalize pain as part of their sport, especially if connected to masculinity and the athletes are men [38]. Participants, moreover, considered themselves ‘reasonably healthy’ at the time of the interview, i.e. none of the participants had an illness identity and exercised for therapeutic purposes. Some of them had some minor ailment, which they rehab-trained for ‘on the side’ but which did not affect their self-image. All of them regularly (3–4 times a week on average) worked out at different recreational gyms, both independently and participating in gym classes (see overview of participants in Table 1). The participants are therefore considered representing the norm of regular active gym-users as recreational athletes. People who volunteered to participate turned furthermore out to be quite experienced and have worked out at the gym for some time. As a rule, they combined different types of exercise in their training routine (for example spinning and endurance-oriented weight training). Some of them were even involved as personal trainers or instructors of gym classes. In these cases, however, the analysis was solely done on interview material in which they talked about themselves as recreational athletes, to keep the focus on ‘normal’ gym-users.

**Table 1:** Overview of included participants

Anonymized name	Age	Number of work-outs per week	PT and/or instructor
Linnea	27	4–5 times	X
Victoria	29	4 times	X
Kristin	28	4 times	
Sara	30	4 times	X
Lisa	31	5 times	
Elin	31	3 times	
Kalle	33	4–5 times	
Jessica	35	4–5 times	
Malin	35	6–7 times	
Pontus	36	3–4 times	X
Josefine	40	4–5 times	X
Hanna	43	2–3 times	X
Rasmus	50	5 times	
Susanne	50	3–4 times	
Nicole	51	3–4 times	
Karin	57	3 times	X
Ebba	59	4–5 times	X
Ove	61	3–5 times	
Gunn-Britt	61	4 times	
Eva	65	1–3 times	
Jonas	66	3 times	
Aki	66	3 times	
Bosse	74	5 times	X
Lotta	76	5 times	

Attended gyms cover a range from national chains to local enterprises and are located in a commuter town close to the Swedish capital. All gyms in this town were approached with a request to recruit participants and eight of them agreed to inform their members. Moreover, a note about the study was published in a local newspaper. 34 people volunteered to take part in an interview and 32 of them, fulfilling the inclusion criteria for participation, were interviewed. As theoretic saturation of data was achieved after 24 interviews, only these were included in this study.

A qualitative approach using individual in-depth semi-structured interviews for data collection [55] has been chosen and preferred to collecting data via verbal questionnaires like the McGill Pain Questionnaire [56]. This is motivated by the study’s explorative nature and openness to unexpected, even positive notions of pain, which medicalized pain scales in general and the mentioned questionnaire in particular are not able to provide. Interviews took place in separate rooms at gyms, the local library, people’s homes and the university department, depending on participants’ choice of a safe, familiar and convenient environment. These interviews lasted between 28 and 85 minutes (most lasting between 40–55 minutes). They were conducted using an interview guide, starting off with associating freely on pain in connection to exercise, followed by questions concerning experiences and perception of pain in a gym environment, feelings about pain, different kinds of pain considered relevant at the gym, reflections on the role of pain in training in general, the relationship of pain to one’s personal motivation and experience of exercising and pain as an identity-relevant phenomenon. Moreover, prompts such as the slogan ‘*no pain, no gain*’ were presented. The interviews were recorded and transcribed verbatim.

It needs to be pointed out that pain other than physical, i.e. of a more mental or social quality, popped up at times while talking about physical pain. Usually, these types of pain needed to be specifically interrogated by the interviewer to be considered and will be discussed elsewhere as their consideration would exceed the scope of this article.

### Analysis

The analysis was based on inductive qualitative content analysis [57] yet undertaken from a hermeneutical stance. In doing so, the analysis could capture not only explicit manifest but also implicit latent content, hence, both “what the text [manifestly] says” and “what the text [latently] talks about” [57, p. 106].

The hands-on analytic process comprises of three sometimes overlapping stages: On the coding stage, meaning units were identified and labelled. These units could differ significantly in size, ranging from words to parts of complete or even several sentences, which can be seen in the result section’s quoting style. On the following stage, codes were grouped into categories, which then were probed regarding their meaning by asking “what does X mean” with “X” being a category (such as muscle soreness). This meaning was derived by going back to the codes and to the data that signified the category with the intention of finding characteristic traits on a latent level. On the final stage, overarching themes were identified while continuing the process of capturing the latent meaning of pain in exercise by identifying the characteristics of the themes (such as impediment) as emerging from their contributing categories. The results were validated in discussions with colleagues who are experienced qualitative researchers.

## Results

“There is a pain that you shouldn’t have and then there is a pain that might be a little welcome. (…) One that furthers you and one that doesn’t.” (Josefin).

Interviewees differentiate between three kinds of physical pain: the good pain of enhancement, the bad pain of impediment and the ‘both and’, neutral pain of acceptance. While the first two kinds describe direct, linear understandings, the last one represents a more complex, composite understanding that balances good and bad characteristics of the pain in question.

### The direct, bad pain of impediment: safety first

The theme of negative, impeding pain is present in all participants’ accounts and always comprises acute damage-related pain and for some even excessive muscle soreness.

All participants present acute damage-related pain as a limit that should not be passed during the work-out in order not to endanger one’s capacity. Even if a sort of pre-stage may be described that “feels wrong” (Malin), indicating that “the body is not content” (Susanne), and can be interpreted as a warning sign for incipient damage, participants usually talk about damage done as a lurking threat. As Malin puts it: “you want to be, feel exhausted, but not ‘end up in hospital’ exhausted. (…) I don’t do it [exercise] in a way that the body would feel bad afterwards”.

The damage-related pain occurs abruptly yet has lasting qualities as a continuous pain that cannot be safely outwaited during the ongoing exercise session. It is this kind of pain, described as e.g. “intense” (Aki), “brutal” (Malin), “sharp” (Nicole) or “stabbing” (Kalle), that is understood as a setback regarding one’s current and longer lasting physical performance/health and impede future exercise:

Rasmus: They [referring to a picture of two people in hospital beds, captioned ‘that was a great workout’] have sustained some type of injury (…) You shouldn’t end up like this, this will only spoil exercise in the long term, because they can’t work out again tomorrow and the day after tomorrow.

The hindrance of one’s capacity for further exercise, i.e. an impeded exercise functionality, is presented as the major reason for this kind of pain being labelled “bad” (Kalle) or “negative” (Hanna) by regular gym-users. Moreover, gym-users exposing themselves to this kind of pain or increasing the probability to do so are deemed irresponsible: “Then you have run down your body. Then it’s actually a question if you deserve it [the body]” (Hanna).

Damage-related pain is considered to be caused by a movement that is exaggerated (e.g., overwork or overstrain) or wrongly executed from a technical point of view (e.g., as an accident or by compensating unevenly when tired). Consequentially, this type of pain calls for immediate action and is most often dealt with by stopping the exercise, reducing weights or adjusting one’s technique. Here, the border is crossed between good and bad effort/strain or between good and bad control/ technique, which are characterized by the limit of one’s potential capacity and performance. These two mark the border between good and bad pain, at which the safety of exercise is compromised. Navigating this exact border is a major task for all as Hanna describes: “you have to learn the difference, to distinguish between the positive pain and the negative [pain] (…) you shouldn’t dislocate a shoulder, but it may burn in the shoulders”.

The perception of the border between hazardous and non-hazardous pain and actions taken can vary considerably between participants. Some stop at once and decide “Me like ‘this may probably damage my back’, I just ‘no but I don’t want to do this.’” (Malin). Others may choose to continue as if nothing too serious has happened: “I sprained pretty badly at my high intensity pass once (…) But I shit on it and thought it’s just sprained and that’s what it was. Then I taped it and continued. It was stupid because after 15 years I needed an operation.” (Ebba). It is hence the perception of danger that characterizes the connotation of acute damage-related pain as negative discomfort.

Muscle soreness, the other kind of negatively connoted pain, is a pain that may tip over into a negatively connoted discomfort when it becomes excessive after crossing the border of good effort. At this point, a hazardous impeding impact comes to the fore:

Kristin: it is difficult to grade like ‘good pain’, ‘bad pain’. But I would probably say (…) if you move [your shoulders] backwards and you feel that it’s a bit tense (…) a bit tired, then it’s good. But in case it starts to become a kind of movement impediment, (…) you can’t go up the stairs, you can’t sit down properly. Then you have pushed too much.

Excessive muscle soreness owes its negative connotation hence to its impeding impact, which in this case concerns daily routines outside the gym, i.e. an everyday functionality, for example emphasized as “It should work in everyday life” (Rasmus). Excessive muscle soreness endangers this functionality and is hence no longer characterized by safety. Perceived limits for excessive muscle soreness vary, again, individually, ranging from Elin’s unwillingness to experience sore muscles at all (“I hate sore muscles”) to Rasmus stating that getting real bad muscle soreness in the gym is “very difficult”, something he only accomplished once:

Rasmus: Two guys from the hockey team showed up who are both considerably bigger and stronger than I am. And then you sort of end up in not wanting to give up, in a competition. (…) The point [of intensity] at which I would get a good workout, that is probably where they start to warm up. After this work-out, I couldn’t walk for about a weak.”

Here, Rasmus describes how he, against better judgment, attempts to defeat someone he cannot defeat, driven by an overambitious attitude that made him act irresponsible, even stupid towards his own body by endangering its safety. Or as Bosse puts it “Big will and small brain.”

A convergence between acute damage-related pain and sore muscles can be assumed as the latter is commonly explained as damage in terms of tissue de- and reconstruction: “the body, it has emptied everything [resources] and now they need to be restored. I’ve broken down muscle fibers (…) and now they are rebuilt” (Lisa). In both pains muscles are concerned, which are generally regarded tenacious and safely improvable. If, however, these pains exceed certain limits, the assumption of a serious, degenerative damage (beyond safe reconstruction) appears to take over, which causes the negative, impeding discomfort. The time frame is a different one though. While damage is expected to appear acutely and last longer in damage-related pain, muscle soreness-related overstraining causes damage that emerges subsequently and is relatively short-lived, which is often understood as reassuring with regard to safety concerns: “it will pass (…) it’s not that the body indicates there is something actually wrong” (Susanne).

People who highlight this understanding of pain most likely reject the ‘*No pain, no gain*’ dictum, as pain in this case is not assumed to provide a gain.

### The direct, good pain of enhancement: ambition first in a quest for performance

Both muscle soreness and effort burn have been very clearly addressed as good kinds of pain.

Usually, appropriate muscle soreness is not regarded negative, quite on the contrary:

Ebba: “sore muscles, that’s the best form of pain (…) because then I know I’ve done something, I have really struggled and used new muscles”.

The positive assessment of its cause and effect in conjunction with its ephemerality (indicating safety), contributes to the positive assessment and dismissal of discomfort. Muscle soreness is here not only a statement about having worked out but a direct translation of the extent of one’s commitment to exercise and the correct intensity of its execution (one’s effort) into a pain-mediated message. This level of ambition represents a judgement of the rightness of one’s exercise as a desirable way of conduct that is not only technically but also morally right. Thus, muscle soreness delivers “a receipt” (Karin) or even “a reward” (Lisa) for this rightness of successful exercising:

Kalle: I think muscle soreness is quite nice (…) it’s almost a little nice to wake up and feel you have sore muscles, because then you know that you have made an effort, possibly in the right way.

This “right way” implies for some to do something extraordinary – ”I have done something beyond” as Karin puts it – that, however, does not cross the line to damage and therefore still remains within the limits of a safe work-out, conducted by a responsible gym-user. As such, this right exercise indicates the idea of increase and, consecutively, (self-)enhancement due to the right effort, as the reconstructed tissue is not only ”new” (Lisa) but rather “more” (Susanne). There is, hence, a congruence between ambition and enhancement with pain symbolizing an (expected) increase in strength as central to sore muscles. This betterment also becomes apparent in the following quote:

Lisa: I love it (…) when I have sore muscles, I almost feel a bit fresh, a bit newly showered. (…) I think it’s like when you’ve washed your hair and it’s completely fresh, for me, it’s like I feel a little better. (…) Because it somehow also becomes like a reward, that I was reminded that I actually did this yesterday instead of having a day where I just lay on the couch.

The comparison with being freshly showered shows the intended effect of self-improvement and the satisfaction that comes with it while simultaneously pointing to its everyday context, constant need of iterative maintenance and basic level of self-care – or as Malin puts it: ”then I feel that I know that I am alive”, presenting muscle soreness as a confirmation of basic conscious existence.

Effort burn is described as “that burning pain when you’re working out” (Lisa) and often positively appraised as “simply good” (Malin), “a reward” (Lisa) or even “a pleasant pain” (Rasmus), i.e. a pleasurable experience instead of a negatively connoted discomfort. This also becomes apparent in some participants’ refusal to address the experience as pain at all: ”it’s uncomfortable, but not completely, it’s not pain for me but more like uncomfortable, the muscles may hurt but in a positive way” (Sara). Usually, however, the burn is experienced as an acute, short-term discomfort.

Effort burn is understood as indicating positive muscle fatigue as the muscles’ regular and intended answer during a work-out, which is considered a necessity to achieve the aimed-for effect of enhancement in terms of increased muscular strength: “what I do when I’m completely exhausted and do one more rep or two more reps (…) that’s what causes the improvement (…) until you get really tired, it’s only a transportation route” (Rasmus). Effort burn represents thus the right high intensity of and great commitment to an exercise that is conducted aspiringly, on the right level of ambition “when you push a little extra” (Linnea) with the intention to get “beyond your comfort zone” (Aki) while working-out, instead of doing “routine exercise”, characterized as “you are comfy (…) a bit lazy” (Linnea). This characterization of the burn as an immediate, positively framed response is also represented in experiencing it as a joyful kick, making people feel ”super-duper alert” (Malin).

Another of the burn’s important characteristics is its safety, as represented in its perception as short-lived and involving muscles equilaterally. Achieving the burn as such a safe signifier of the right, ambitious effort is often ascribed to mental strength: “a lot depends on the brain” (Malin). Even this kind of pain owes its positive connotation therefore in part to an exercise’s responsible, reasonable execution, which is often addressed as ‘listening to the body’:

Aki: if you’re out running (…) you can set a goal, ‘now I’m going to run six kilometers’, but then you manage only three, that’s it, and then you listen [to your body] (…) I think like 40 years ago I might not have accepted, instead that devil would have come and told me ‘you have to finish this round, whatever it takes’ but today it’s not like that, I’m listening.

It is notable that several participants mention that they most likely reach this level of effort only while exercising with somebody else: “It’s rare that I come to that [level] (…) when I train alone (…) [With someone else] you feel pushed in a positive way” (Linnea). Despite the burn’s characterization as a signifier of individual ”frontal lobe” (Elin, using a figurative expression for willpower), group dynamics appear hence to push people to persevere more, experiencing larger resulting satisfaction: “I’m happy after a such a military session, which has kind of pushed me to just do even more” (Malin).

Comparing muscle soreness and effort burn, the former appears to function subsequently while the latter does so immediately, proving in both cases self-discipline and conquest, i.e. the right, responsible level of ambition. Both kinds of pain show an ongoing enhancement in performance and represent an appreciated, agreeable experience that is not necessarily understood as discomfort but rather well-being. While this enhancement appears to be a short-lived exhibit of potential and pushing boundaries during the burn experience, a promise of a “more”, muscle soreness rather represents the painful tissue reorganization that is understood to consolidate this “more” of pushed bodily limits of performance, which have been exhibited.

People who predominantly adhere to this notion of pain most likely embrace the ‘*No pain, no gain*’ dictum, based on an understanding of pain as a primarily physical experience during the work-out that is supposed to lead to a gain in terms of an enhancement.

### The composite, neutral pain of acceptance: discomfort on demand

All of these pains may also be understood as rather neutral, acceptable pains. The neutrality results from negotiating the different characteristics of pain and combining them into a composite assessment rather than to ‘take sides’ in terms of labelling the pain ‘good’ or ‘bad’. This neutral understanding usually does without the appreciation of a ‘no pain, no gain’ attitude: ”it must hurt to get somewhere, no, that’s definitely not the trail I’m following”(Nicole). Acceptable pain is, moreover, generally connected to a diminished emphasis of imperative enhancement as aimed-for outcome, or as Ove says: “my training is based on it being an expression of my well-being (…) rather than that I’m weak and I’m going to damn well show that I can get stronger”.

One of the mentioned pains is muscle soreness, as described by Susanne: “If it would be very difficult for me to walk because I have muscle soreness in my legs (…) just keep going, it will get better. If your blood circulates you won’t be in as much pain (…) it sure is not comfortable, but no danger”. She directs attention to the ‘both and’ combination of acknowledged discomfort with safety as indicated by the short-termed, not impeding characteristics of uncomfortable consequences. Such a safety counterbalances negative discomfort in a matter-of-fact ‘stand-off’ between muscle soreness’s two sides, which may even include a slightly positive function as an incentive to train (to increase blood flow) even if it is presented as neutral.

The description as “a necessary evil” (Aki) elaborates on that by assigning an element of inevitability to muscle soreness, which contributes to balancing discomfort. Inevitability indicates ambition in terms of achieving the goal of an expected benefit. By connecting ambition to discomforting pain, its perceived discomfort is counterbalanced and judged as something that exists but is worth enduring, which is often addressed in a pragmatic way as unavoidable, safe and thus acceptable:

Aki: it [muscle soreness] passes after a couple of days, but no, I don’t have a problem with muscle soreness, I just know that it’s coming (…) that’s just the way it is, [the fact] that I experience muscle soreness, it’s proof that I have carried out the exercise.

The benefit of this pragmatic approach consists mainly of body maintenance: “there are a lot of people in my age group in here [gym] who have had knee surgeries or shoulder surgeries, you name[-it] (…) I guess I come here to try to avoid ending up in that situation” (Aki). Well-being exists but is in this case a consequence of having worked-out rather than something that is experienced while working-out: “it feels good when you have completed the entire program as planned” (Aki). This cluster of characteristics – uncomfortable yet safe, inevitable, thus acceptable – is a commonality between muscle soreness and effort burn, in cases when the burn is not embraced and longed-for as enhancement-related but rather represents effective exercise successfully aiming at body maintenance as well.

The same characteristics can also be recognized in a neutral version of acute damage-related pain that is perceived as acceptable. This is the case if the damage is viewed as safe in terms of inevitable concerning the type of executed exercise as well as connected to minor/short-term damages. Here, the damage is not so much a sign of an overambition but rather of a general commitment to this type of exercise. It is noteworthy that acceptable damage-related pain is usually observed as related to exercise other than working out at the gym (e.g. in Thai kickboxing or handball): “some pain must of course be expected in some way if you box and Thaibox ‘Yeah, I gonna be blue on the legs and gonna get punched” (Susanne).

A slightly different version of the acceptable damage-related pain can be observed if the damage in question is chronic (e.g. osteoarthritis or a persisting impairment of a joint after major injury) or long-term (e.g. an effect of a muscle-rupture). In addition to safety and ambition-guided inevitability, differently timed kinds of discomfort matter in this case. Acute discomfort when experiencing pain during exercise is negotiated with delayed discomfort before and after exercising. Increased discomfort before exercising may be deemed a signifier of the need to engage in exercise. Factual or expected diminished discomfort after exercising, compared to an expectable discomfort without exercising, is regarded a clear benefit, even if it means enduring discomfort while working out.

Interviewer: What happens if you don’t exercise? (…) do you notice that it affects your pain in any way?

Gunn-Britt: The osteoarthritis? Not while I’m not exercising, but when I start again, it hurts more. So I need to keep going continuously to keep it in check.

Interviewer: But you say that you are actually constantly in a bit of pain when you exercise. You feel it in your knees. (…) But you still exercise. How do you motivate yourself to anyway?

Gunn-Britt: That I’ll experience terrible pain if I don’t, I know that. Because I’m that old. If I stop now, I’ll never get started again. Here we go. All the way to the end. That’s it, that’s the plan.

Hence, both kinds of delayed damage-related discomfort can be connected to maintenance, namely of both body functionality and of exercise routines, and appear to even include the implicit and delayed well-being of avoiding pain in everyday life. They function in practice as an instrumental incentive to engage in exercise. All things considered, even if there is still a negatively connoted, yet inevitable and safe, thus acceptable discomfort, this type of damage-related pain also features an exercise-supporting effect. It thereby gains a positive tinge for this group of active, normal gym-users that may exceed the acceptability of a ‘necessary evil’ characterizing the other kinds of acceptable pain.

Taking this understanding of pain as a basis may also lead to approving the ‘*No pain, no gain*’ dictum, which, however, may be interpreted in a different way. In this case, pain may refer to the process of overcoming (often mental) barriers to go to the gym and work out properly, whereas gain may rather represent the contentment of following through with one’s (potentially health-maintaining/promotive) work-out plan, instead of increasing one’s performance.

## Discussion

The main findings indicate that overall, participants consider physical pain a messenger, a way for the body to express itself. Listening to the body talking through pain is a necessity for these active, ‘normal’ gym-users. Assessments regarding the level of safety, ambition and a resultant discomfort in different time frames are negotiated and lead to different understandings of pain as well as the ‘*No pain, no gain*’ dictum. Whereas direct understandings of pain are always connoted as either good or bad, representing the enhancement or impediment of performance, exercise and everyday life, assessments on which these two viewpoints rely may also be merged. In this case, differently assessed characteristics ascribed to the same pain are negotiated and weighed against each other. The resultant pain of acceptance represents a combined, more complex understanding with rather individual outcomes. As such it is a rather messy, or ugly, business as its interpretation is depending on every gym-user’s comprehension of acceptable levels of safety, ambition and discomfort, which cannot be standardized and may or may not work in an instrumental, exercise-promoting way. For these gym-users, the ‘*No pain, no gain*’ dictum might not describe a necessity for enhancement but a barrier for contentment, which in both cases needs overcoming.

### Pain and exercise: a straightforward relation?

When compared to earlier research, there are overlaps as well as less well-established, new results.

In general, the findings confirm that all gym-users agreed on expecting pain to be a part of the gym experience [cf. 8], although assessed very differently by different participants [cf. for example 44, 45]. Moreover, participants underline the implicit task to get acquainted with and manage the boundary between good and bad pain as a main task for the active gym-user [18, 41]. Not least, even in this study, impending damage-related pain is generally considered negatively connoted [cf. e.g. 23, 24, 29], despite its medically pronounced functionality as a warning [28], as is pain’s negative assessment when linked to excessiveness [7].

Some participants may though understand muscle soreness and effort burn as positively connoted kinds of pain, which has been described before [17, 32, 41]. This understanding is reflected in them promoting the enhancement-focused understanding of the saying ‘*no pain, no gain*’ [28, 44], which underlines a well-known description of gym culture in general [cf. 9, 34]. Thus, participants voicing this understanding may be likely to confirm the ‘old’ core values of bodybuilding as well: intense exertion-oriented muscle training, asceticism and competition [5]. This indicates not only the transition of exercise understandings stemming from professional sports [28] and military training [58] into the realm of everyday recreational exercise but also a similarity between recreational athletes and those engaged in different types of strength athletics [cf. 32–35].

Especially (anaerobic) pain experienced during work-outs is enjoyed and embraced by strength athletes [cf. 32, 34] and some of my participants alike. One considerable difference is, however, worth mentioning: The latter refer exclusively to effort burn in this regard, i.e. the burning of the muscles when reaching/crossing the anaerobic threshold, compared to the former, for some of whom the phenomenon of the pump, i.e. the swelling of muscles due to exercise-induced increased blood flow, is depicted as the pleasurable experience [cf. 32, 33]. The pump enjoyed by strength athletes appears to represent a more immediate pleasure as it is the muscle swelling in itself that is enjoyed, not a bodily sensation as a representation of an enhancement yet to come. Moreover, and despite one mentioning of a “seductive” pain [36, p. 186], the accounts of normal gym-users lack the erotic sensual qualities, which some strength athletes may associate with the pump. Therefore, both pleasurable experiences, although related due to their connection with anaerobic exercise, appear to be qualitatively different. Then again, the experience of an exercise-related rush similar to the consumption of drugs, can be observed in both groups [32], as is the general connection between positive pain on the one hand and achievement, self-mastery and (often) responsibility on the other [34, 35].

Curiously, sore muscles are not as praised by strength athletes, compared to the sub-group of my participants highlighting positive pain. This might indicate a distinctive feature of the group of normal gym-users as the same appreciation – even referred to as “glorious pain [17, p. 295] – shows in Lev’s study with a similar target group. It could be argued that the group of normal gym-users is more in need of confirming successful exercise sessions as they not necessarily aim for visible muscle growth, which could provide sufficient correspondent feedback. What has, however, not at all shown in the accounts of my study participants is the credit both strength and recreational athletes have been giving to a process of learning and socialization into the gym culture as that which greatly enabled their ability to enjoy both sore muscles [17] and anaerobic pain during exercise [32]. This lack might be attributed to this study’s focus on current understandings of pain. The only learning process that was hinted in the data material was related to getting to know the border between good and bad pain, but not an appreciation of a certain type of pain.

This study hence widely confirms existing research regarding the landscape of gym-related physical pains, which may be clearly and straightforwardly labelled as ‘good’ versus ‘bad’. Provided that active normal gym-users imbue them with the meaning of enhancement and impediment, respectively, the former understanding can be easily linked to the transformativeculturally ingrained capacity of pain in western societies whereas the latter to its deconstructivecounterpart [49]. To impede pain’s deconstructive capacities (i.e. occurred damage and excessiveness) and enhance its transformative potential (i.e. appropriate intensity, control, commitment) appears then to be logical connected to a health promotion characterized by demands for control and self-realization on the one hand and pleasure and release on the other [6].

Here, a fine line between discomforting and pleasurable pain exists that may serve as guidance concerning the individualized task to negotiate the boundary between too much and too little, at which both safety and ambition may be actualized and the responsible health-conscious citizen emerges – even if individual boundaries may vary greatly. It is, however, imminent to the principle of health promotion that this boundary between good and bad pain is supposed to be constantly pushed forward due to committed body work [cf. 48]. Hence, the insecurity concerning a possibly insufficient production of an ever-fitter body and the anxiety regarding a related potential to miss out on consumable sensations may only temporarily be kept at bay in this never-ending process of becoming one’s ‘best self’. This indicates a relation between production and consumption, which may be addressed as ‘production (of the body) for consumption (by means of the body)’ [cf. 51], highlighting a body relation, which – if successfully accomplished – may be regarded as both instrumental and symbolic [cf. 35].

In cases when positively labelled pains are understood as pleasurable rewards – i.e. when muscle soreness and effort burn are understood as exclusively good – gym exercise may indeed be considered the perfect match of health promotion’s ritualic ingredients pleasure and control. Performing a routine of disciplined, yet all-in energy-releasing work-outs represents the productive consumption of health promotive resources at the gym and consumptive production of the ever-optimizing, constantly health-enhancing self [cf. 47] and thus merges those seemingly opposite demands. This situation has been labelled “production within consumption” [35, p. 220], describing that the body is shaped/produced by utilizing the frame of a heavily commercialized global gym enterprise [4]. Judging from my data, it could though just as well be addressed as ‘production as consumption’ as the process of production provides in itself the pleasurable sensations sought by citizens of a consumer society [cf. 53], inextricably intertwining both processes in this case. This is most obvious implied in the description of the rush [cf. 32] but becomes also apparent when muscle soreness and/or effort burn are labeled as “pleasant” or “nice”.

In the longer run, a pitfall may occur, however. The infiniteness of the process of becoming ever more fit and healthy [cf. 51, 6] perpetuates painful production, whereas the pleasurable sensations experienced during that process may simply wear off. Exercise may then no longer be deemed enjoyable but a duty to keep up the fitness level. When entering this treadmill, good pain may no longer be experienced as pleasurable but become ordinary or even ‘bad’ in that it fails to function as a receipt or reward for doing the right thing anymore. In fact, they may disappear altogether (in the case of muscle soreness) and in doing so represent adaption, a plateau with regard to experience and performance, which may easily be understood as frustrating by the sensation seeker. Experiencing this plateau may feed into the never-ending process of fitness/health production in that it may be translated into a need to raise the level of exercise intensity. This may, if done repeatedly, fuel potential obsessive tendencies in health promotive exercise [6]. A once positive, transformative pain might hence convert into something that rather works in a negative way and becomes a representation of pain’s deconstructive capacity [cf. 49], even if it not necessarily may be experienced in that way by the individual exerciser.

Proceeding to study results that are less highlighted and explored in detail in previous research, the ‘ugly’ kinds of pain come into focus. Traces of pain, which may be termed ‘acceptable’ following the description of my participants, are mentioned in the literature [36–40, 42, 43], yet without really emphasizing a supporting function. Existing research, however, does not differentiate and explore the various related kinds of pain and their contexts in detail. As an example, Arli et al. [44] state that “some respondents understand there has to be some pain to gain” [44, p. 104], which the authors connect to an appreciation of the saying ‘*no pain, no gain*’ by “keen exercisers” [44, p. 112], but without further elaborating neither what kinds of pain are involved nor what meaning the ‘*No pain, no gain*’ dictum might have for those who appreciate it in this context. In this study, an appreciation of an enhancement-focused understanding of the dictum did not show in those who have a more pragmatic, accepting understanding of muscle soreness, effort burn or certain kinds of damage-related pain, whereas they may appeal to a contentment-focused understanding.

While an acceptance of sore muscles and the burn appears to be less surprising (as e.g. hinted at in [40]), damage-related pain is usually considered a barrier to exercise, not a supporting influence [see e.g. 23–25]. Here, a more differentiated view of the addressed kind of damage as well as its timeframe appears to be recommended. It is, moreover, notable that different kinds of pain are not per se viewed as ‘good’ or ‘bad’ but in relation to the meaning they are imbued with as representing enhancement or impediment – or as Josefin pointed out in the initial quote, there is a pain “that furthers you and one that doesn’t”. Therefore, the interesting question is not what kind of pain people experience but what meaning this specific pain is given by them. Seen in this light, all kinds of pain are ‘ugly’ as their meaning is not universal but individually assigned.

From the point of view of health promotion as a ritualistic practice [6], different acceptable pains clearly represent disciplined control and a self-realization that is not part of the work-out but delayed until afterwards. While in the gym, emphasis is put on the notion of productive control, instead of pleasurable release, which also manifests itself by coinciding the experience of well-being with the completion of a work-out, i.e. emphasizing the release of a goal-focused practice as the pleasure awaiting the responsible, health-conscious individual after work. The transformative capacity of pain hence predominates as a motive for maintaining an exercise routine while its deconstructive side is attached simultaneous importance as a balancing influence on just how productive this exercise routine is assumed to be [cf. 49]. In contrast to a pain that is understood as impeding or enhancing, one that is acceptable does not relate to one of pain’s capacities only but to both at the same time, thereby underlining the need for control and discipline to hold its unproductive side and potential dangers awaiting the body and self at bay. Even in this case, consumption facilities are of course employed to produce a certain body, thus indicating the existence of a ‘production within consumption’ relation [cf. 35]. For people emphasizing acceptable pain, however, the predominant relation between consumption and production appears to be represented by a ‘production for consumption’ relation, considering that both the pleasure of well-being and the self-realization of the individual are postponed until after the time spent exercising in the gym [cf. 51, 53]. Here, consumption takes on the role of an aim for a productive work, which is responsibly carried out. This relation is also indicated by an emphasis on body maintenance, revealing a clearly instrumental connotation and hinting at the possibility that the fear of missing out on new sensations may no longer be these people’s main concern. This might be a source of contentment but also of new anxieties and insecurities in liquid modernity.

Finally, it can be stated that in all its variations, pain can thus be considered as something meaningful, not least regarding its instrumental, productive value for health promotion. The real danger would therefore be a pain that is meaningless, without a message and hence to no avail, not even applicable as a warning or signifier of a (manipulable) damage.

### Practical implications of characterizing exercise through pain

In this section, I return to the literal gym floor to dwell on practical implications of different understandings of pain in this environment. In doing so, I approach the question of how to deal with different levels of allure of pain-related exercising as indicated in previous research [34, 44, 45] from a norm-critical point of view.

If universal meanings of pain are assumed and suggested by gym-users they are normalized and turned into exercise-related expectations [cf. 46], especially if the gym-user in question also leads gym classes, hence taking on the role of a (quasi) expert. Advising caution concerning the construction of these expectations appears thus to be a first practical implication.

Moreover, characteristics ascribed to pain may turn into characteristics of exercise itself as the latter appears to be inextricably intertwined with the former [cf. 44, 41]. Focusing on its normative core message, good exercise may consequentially be understood as a safe, disciplined, arduous endeavor rewarded with pain while bad exercise will be either painless but ineffective or outright harmful, resulting in more or less delayed impediment either way. There are two potential consequences. (A) While good exercise, thus understood, clearly appeals to some, it surely may put others off. Thus, pain may serve both as a facilitator and a barrier to exercise. This leaves professionals with the task to figure out which of these understandings applies to the individual in question and not least provide activities characterized by the proper amount of the right kind of pain. (B) If due to its normative frame the communicated alternatives regarding exercise consist of experiencing either pain or pain (immediately or delayed), some people will most certainly abandon the idea of this kind of health promotive physical activity and try to postpone the inevitable, maybe even more so if the good pain is praised too much and the enhancement-focused understanding of the saying ‘*no pain, no gain*’ is emphasized. It can therefore be assumed that the way exercise in general and certain kinds of exercise in particular are presented should be chosen with great care. In addition, it might help to offer the contentment-related notion of the ‘*No pain, no gain*’ dictum as de-dramatizing alternative understanding, to broaden the spectrum of what is presented as ‘normal’ in the gym.

In this context, the difference in emphasis on high versus low intensity exercise in the gym (as observed while visiting different gyms to recruit participants) is worth mentioning: While high intensity exercise is a self-evident, even eponymous part of gym classes, low intensity exercise is not and is very rarely mentioned at all. This is at least surprising, given evidence that low intensity exercise may, in fact, be quite efficient to accomplish health promotive effects [see e.g. 59, 60], even when compared to high intensity exercise [61] even though high intensity exercise undoubtedly has the potential to greatly improve people’s capacity to exercise and promote certain types of health [62]. Observing the different standings of low and high intensity exercise suggests an ascribed status hierarchy, in which high intensity corresponds to a high(er) status and may even be associated with greater skillfulness, all indicating high desirability. This effect may be increased if furthermore certain gym-users are labelled representatives of high performance and skillfulness, turning them into exemplary athletes, i.e. when norms in terms of ideals (of exercise, of exercisers) are invoked instead of representing ‘the usual’ as a less judgmental take on normality [63]. In consequence and in accordance with the study results, praising ideals may turn out to be demotivating for those not abiding by the enhancement-focused ‘*no pain, no gain*’ directive while others may be discouraged if and only if it is avoided to accentuate these precise desirably painful exercises and its idealistic connotation. Communication includes hence the act of balancing expectations and broadening normal understandings, turning tailoring communication to the aimed-for gym-user into a decisive demand for the promotion of exercise as a potential promotion of (certain types of) health.

### Why pain aversion in the first place?

As this section has so far undoubtedly constructed the figure of the not so pain-avid individual who challenges the appropriateness of the ‘*No pain, no gain*’ gym culture as a health-promotive environment, I want to finish with some reflections as to the reasons of this individual’s pain avoidance.

The obvious answer would be to point out the culturally ingrained prevalence to avoid a pain, which in a gym-context might be understood as (self-)inflicted torture [cf. 39]. This understanding of pain may easily be connected to medical efforts to eradicate pain [49] and universalized by relating it to the psychoanalytic pleasure principle [64]. Here, avoiding pain assumes the shape of pain disposal.

Then again, there might be a less obvious candidate for explaining why people shy away from pain related to Bauman’s description of the sensation seeking consumer in liquid modernity [53]. In a neoliberalized, consumist society that emphasizes consumption as the hunt for pleasure and suggests that everything is available on the market, some people may consider the need to produce in terms of working (out) for a body in a discomforting way a frustrating impertinence. This attitude is well-documented and usually interpreted as a lack of discipline, as depicted in this comment of personal trainer “They [some customers] want the body but they just can’t handle what it takes to get it” [in 45, p. 170, see also 54, 34]. In this case, avoiding pain might indicate a pain-related displeasedness.

Whereas people not going all the way to pain may still be physically active, a final attempt to explain some people’s aversion of exercise-related pain might be related to complete absence. In our healthistic times [cf. 65], moralized demands to be physically active for the sake of health – along with the shame of not complying – are quite obviously particularly tangible for those who do not exercise. Hybholt [66], for example, compared motives for exercise and could show that while physically inactive women would justify potential exercise with reasons related to health, physically active women no longer do so but state other reasons – just like bodybuilders not necessarily appear to base their practice on health reasons [cf. 33]. Adding the imagined pain of exercise to the real pain of shame – a shame that might not automatically vanish with the onset of exercise as stigmatization of the not-quite fit(-ting) body is a reality in the gym [67] – may lead to an exhaustion resulting in probable inactivity. This approach to pain aversion might hence rather be called pain fatigue due to a sort of pain overload. Here, issues of power are most obviously at play.

### Limitations

As usual, qualitative studies cannot claim to be statistically generalizable. Provided that the data material was collected in one town only, results may represent a very local view. On the other hand, this specific town has a high ‘gym density’ with different kinds of represented gym environments, which may attract different demographic groups. Moreover, the town is located at the borders between a rural and urban area. It therefore represents a promising context for a data collection that crosses certain boundaries.

Other characteristics may, however, have diminished the informative value of the study: Participants, for one, were predominantly ‘ethnic’ Swedes. As Swedish (gym) culture does not appear to be significantly divergent from neoliberal Western culture in general, as depicted in the background, the composition of the group of participants is, however, not deemed critically limiting this study’s informative value. If anything, the Swedish proneness towards a more gender-equal gym culture is rather considered an advantage, as it may have facilitated the recruitment of women (see Table 1) whereas the somewhat advanced Swedish focus on the independent, self-realizing, self-challenging individual may set a valid case in point for the experience and role of exercise-related pain in neoliberal Western gyms.

Moreover, younger gym-users (aged 18–26) did not volunteer to participate despite active efforts to recruit them. This may be related to a recruitment strategy, which did not include social media, or could be due to these youngsters not having felt addressed by the topic of the study. It is, though, noticeable that the results of this and earlier studies show certain similarities, which argues for a reasonable representability and quality of this study. As for the young adults, it is moreover noticeable that they have been referred to several times by participants of this study as illustrations of different kinds of pain. Even if participants’ observations are of course not equivalent with personal accounts, they may indicate that the young adults’ experiences may, to a certain degree at least, be compatible with the study result.

### Further research

Further research may focus on different aspect of the research design and focus: One option would be to include potential participants whom this study failed to recruit, to complete the picture of perceived pains in the gym: people with an immigrant background of whatever nature and young adult gym-users aged 18–26. Both groups would be interesting to follow with regard to the pain-related learning process, which has been mentioned in previous research [17, 32] but did not show itself in this study. Another target group, whom I could not attend to adequately in this study (due to a lack of ethical approval), are older adults, who frequent the gym on a regular basis and have a long-standing routine to do so. As they are seemingly underrepresented in research [cf. 9], they could be another target group worth the while in studies to come.

In addition, potential relations between specific aspects of exercise and certain kinds of pain were not investigated, due to this study’s explorative focus on providing a general overview of perceptions, experiences and meanings of physical pain in the gym context. Further research projects could therefore, for example, cater to how participants perceive and navigate exercise-related physical pain depending on their experience level (such as novices versus veterans) or the types of workouts they engage in (such as aerobic versus anaerobic exercise). Moreover, subsequent research projects may change focus from physical to other types of exercise-related pain in the gym and how these are related to one another, or take up questions of identity formation.

Finally, further research could use different or more elaborated theoretical angles to understand pains in the gym and interrogate the pain landscape. In this paper, theoretical resources were chosen guided by a quite basic focus on existing cultural resources for making sense of pain and on health promotion, mostly from a practice-oriented or even pragmatic point of view that only touches on the wider context of health promotion. Other research could here easily elaborate the focus and apply, for example, power-related resources concerning healthistic self-government or approaches of embodiment and body work in the gym.

## Conclusion

Pain connected to exercise is a multifaceted phenomenon including good, bad and neutral kinds of pain that are understood as enhancement, impediment and acceptance. When pursuing the goal of personal health development, ‘normal’ gym-users argue that exercising at the gym means to expose yourself to pain and to do so willingly, even longingly. Refusing to share this understanding may diminish people’s chances to occupy the gym space and, hence, reduce their chances to promote their health. Moreover, pain may turn out to be a facilitator of exercise for some while a barrier to other, calling for differentiated, individualized assessments and communication.

## Data Availability

The datasets used or analyzed during the current study are available from the corresponding author on reasonable request. To protect the participants’ identities and due to the type of consent we received from the participants, the full interview data of this study (transcripts and audio files) will not be made available to the public.

## Declarations

### Authors’ information

Pelle Pelters is an associate professor and senior lecturer in (health) education at Stockholm University. S/he is a sociologist with roots in educational science with a focus on (norm-)critical education and conducting health studies from a critical point of view. Research interests include health as an order of power, its morality and normality, as well as health disparities and the meaning of good health and health-related practices.

## Abbreviations

CIF: Centrum för idrottsforskning (Swedish Research Council for Sport Science)
PA: Physical activity
WHO: World Health Organization
